# Clinicopathological Proficiency in the Diagnosis of Kaposi's Sarcoma

**DOI:** 10.5402/2012/565463

**Published:** 2012-05-30

**Authors:** Louis-Jacques van Bogaert

**Affiliations:** National Health Laboratory Service, Polokwane/Mankweng Hospital Complex and University of Limpopo, Limpopo Province, 0700 Polokwane, South Africa

## Abstract

*Background*. The prevalence of Kaposi's sarcoma (KS), an AIDS-defining illness, has increased in parallel with the HIV/AIDS epidemic. The presence of violaceous skin lesions should raise suspicion of KS. However, especially on dark skin, KS mimics a variety of non-KS skin conditions. Histologically, there is a wide range of expressions of KS and a large number of mimickers. For all these reasons, a HHV-8 immunohistochemically biopsy-proven diagnosis of KS should be the gold standard. *Methods*. Prospective study of 490 consecutive skin biopsies from the general community in the Limpopo Province of South Africa, from April 2010 through December 2011. Results. The clinical discordance rate (over-/underdiagnosis of KS) was 30.5%; the histological discordance rate was 9.2%. *Conclusion*. Because of the magnitude of diagnostic error, both clinical and histological, all clinical lesions suspicious of KS should be biopsied and HHV-8 LAN-1 immunophenotyped.

## 1. Introduction

Kaposi's sarcoma (KS) became an AIDS-defining illness at the outset of the human-immunodeficiency-virus-(HIV-) induced acquired immunodeficiency syndrome (AIDS) endemic and pandemic [1–3]. There has been a striking increase in incidence of KS in both men and women compatible with the evolution of the AIDS epidemic in sub-Saharan Africa [4, 5]. In HIV/AIDS endemic regions such as sub-Saharan Africa, purplish/violaceous skin lesions should arise a high degree of awareness and suspicion of KS. However, the clinical picture of KS may mimic a variety of non-KS lesions. Furthermore, the histopathological expression of KS encompasses a large number of mimics too [6]. Finally, not all KS are HIV-related [3].

Sub-Saharan Africa in general and South Africa in particular are at the epicenter of the HIV/AIDS endemic. Although the prevalence rates vary somehow according to the sources, it was estimated that in 2010 it infected 17.8 percent of the South African population and up to 19.7 percent of females aged between 15 and 45 years [7]. Because of the opt-in policy for testing (voluntary counseling and testing) and the arguable widespread risk of discrimination after disclosure of positivity necessary for access to antiretroviral treatment (ART), an undefined portion of the population's HIV status remains undiagnosed, unknown, or undisclosed [8].

The human herpes virus-8 (HHV-8) or Kaposi's sarcoma associated herpes virus (KSHV) was identified as the infectious agent of all types of KS (classic/sporadic, iatrogenic/posttransplant, endemic/African, and AIDS-related/HIV-associated) [9]. The latent nuclear antigen-1 (LAN-1) of HHV-8 is expressed in all cells infected by the virus [10]. The immunohistochemical identification of HHV-8 by LAN-1 has become the “gold standard” in the diagnosis of KS and the differential diagnosis with its mimics. It has been shown to have a high specificity and sensitivity for the diagnosis of KS [11–14].

Because of prognostic and therapeutic implications of a correct diagnosis, it is recommended to biopsy any skin lesion susceptible of being KS, especially in HIV/AIDS endemic regions. We report an observational cohort of 490 consecutive skin biopsies in HIV-infected and noninfected patients. All biopsies were immunostained with HHV-8 LANA-1; 261 (53.3%) were biopsy-proven cases of KS among a group comprising 248 (50.6%) HIV-infected patients. We investigated the rates of clinical and histological diagnostic proficiency of KS and of its clinical and histopathological mimics.

## 2. Methods

### 2.1. Site and Population

The study was carried out in the Limpopo Province of South Africa. All surgical pathology specimens of public health facilities are centralized at the National Health Laboratory Service in the provincial capital, Polokwane. It serves an essentially rural population in excess of 5 Million. The study was approved by the research ethics committee. All the cases were treated anonymously. 

### 2.2. Data Collection

The cases were collected from April 2010 through December 2011. All the patients were of African ethnicity. The following information was recorded: age, gender, HIV status (when available), ART status, CD4+ T lymphocyte count, site of biopsy, and clinical provisional diagnosis. Only 25 biopsies were referred by specialist dermatologists.

All of the cases were routinely streptavidin-biotin-peroxidase immunostained with diaminobenzidine using a murine monoclonal antibody directed against the C-terminus of the LAN-1 molecule of HHV-8 (clone 13b10; Novocastra, New Castle upon Tyne, UK). The initial histopathological diagnosis was made before LAN-1 immunostaining and corrected accordingly when indicated in retrospect. The clinical and histopathological diagnostic proficiency was expressed in terms of concordance or discordance. Discordance was further subdivided into overdiagnosis (non-KS) and underdiagnosis (KS missed).

Statistical analysis was performed using column statistics, Student's *t* test, and 95% confidence intervals (CI) of proportions. The level of significance was set at *P* < 0.05.

## 3. Results

Out of 490 cases, 261 (53.3%) were HHV-8 proven cases of KS. The rate of documented HIV positivity among the 490 cases was 248 (50.6%). Eighty-one (32.7%) were documented to be on ART and 24 (9.7%) not to be on ART. The average CD4++ count was 259.0 ± 181.6 cells/mm^3^ (median: 214.0; range: 6.0–674).

Among the 261 cases of KS, 161 (61.7%) were known to be HIV-infected; no HIV serostatus was known for the 100 others. Among the KS with unknown HIV status, 52 were males and 48 females. One KS only was HIV negative at the time of biopsy.

Table 1 shows the average ages by gender, HIV status, and diagnosis of KS. There was no statistically significant difference between the groups.

The anatomical site of KS, illustrated in Table 2, was known in 212 (81.2%) cases. The lower limbs were affected in 43.4%; the lesions were disseminated in 29.7%. The head was significantly involved in the HIV unknown serostatus, while dissemination was significantly more common in the documented HIV-infected patients.

Table 3 lists the distribution by histopathological stage. There was no significant difference in distribution of stages between the patients with unknown HIV serostatus and documented HIV-positive cases.

Table 4 illustrates the biopsy-proven non-KS skin lesions according to the HIV status. Pyogenic granuloma and seborrheic keratosis represented close to half of the non-KS lesions.

The histological rate of discordance, shown in Table 5, was 9.2%; 18 KS were missed, and 23 non-KS were overdiagnosed. Fibrous histiocytoma (*n* = 9), pyogenic granuloma (*n* = 5), and seborrheic keratosis (*n* = 8) were misdiagnosed as nodular stage and early patch KS, respectively.

**Figure 1:**
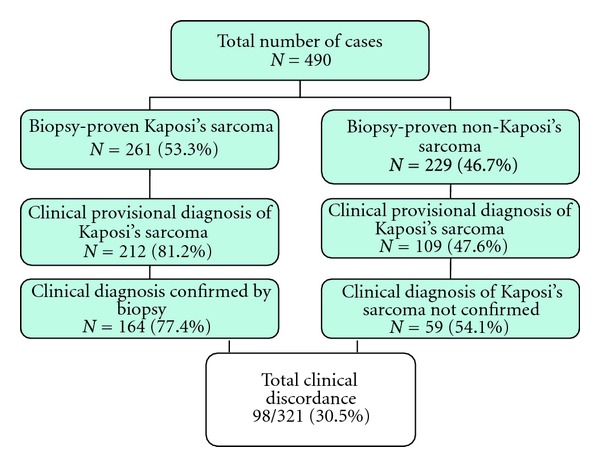
Flow chart of the clinical diagnostic concordance and discordance with histological diagnosis.

Figure 1 shows the flow chart indicating that no clinical provisional diagnosis was provided in 49 (18.8%) biopsy-proven KS, and in 120 (52.4%) of non-KS skin lesions. The total clinical discordance rate was 30.5%; in 48 (15.0%) with provisional clinical diagnosis, KS was overdiagnosed, and, in 59 (18.4%), the diagnosis of KS was missed clinically. Seborrheic keratosis and pyogenic granuloma accounted for half of the total clinical discordance rate.

## 4. Discussion

Kaposi's sarcoma is a multifaceted entity in terms of the population groups (ethnic and gender) it affects, in terms of clinical signs and symptoms, in terms of the body parts it affects (skin, mucosa, lymph nodes, viscera), and in terms of histopathological expression. Traditionally, the six histological variants are the early patch stage, the nodular stage, the plaque stage, the intravascular, the lymphangioma-like, and the angiosarcoma-like type [15]. Recently, more than ten additional variants have been described [16, 17].

In sub-Saharan Africa, KS accounts for about 10.0% of HIV-related malignancies and may be the first manifestation of HIV [18]. As such, the diagnosis of KS may in many instances serve as an entry into the treatment circuit, provided consent is given for HIV testing. Patients with AIDS-associated KS present mostly with advanced disease resulting in high mortality [19]. Although KS regresses under ART, the relatively wide unavailability of ART, and the threshold of a CD4+ count of ≤200 in South Africa as entry criterion into the ART circuit result in a rapid progression of the disease [20, 21]. This illustrates not only the importance of an early and correct diagnosis of the disease but also of the willingness to undergo HIV testing.

Clinically, virtually all pigmented skin lesions are part of the differential diagnosis of KS [22]. In black subjects, the clinical recognition of KS is likely to be even more difficult than in nonblack subjects. This underscores the need of a biopsy-proven diagnosis. Because of the vast spectrum of vasoproliferative mimics of KS illustrated in Table 6, HHV-8 LAN-1 immunohistochemistry has become the gold standard of diagnosis [16, 22–24].

The increasing prevalence of HIV infection in sub-Saharan Africa together with the reluctance to be tested makes it difficult to distinguish the endemic KS from the AIDS-associated variant [5]. The distinction has prognostic implications since endemic KS evolves much slower and less aggressively than the AIDS-associated variant. In a Tanzanian study of 105 clinically suspected KS, 77 (73.3%) were confirmed on biopsy (without HHV-8 LAN-1 confirmation though); 11 (14.3%) occurred in HIV-negative subjects. The majority (63.6%) of the endemic KS cases was in the ≥50 years of age group; the lesions were limited exclusively to the lower limbs, and all were males [5]. In our series of KS with unknown HIV serostatus, there were an almost equal number of males and females, with a similar mean age and distribution by histological stage as the documented HIV-positive KS. In terms of anatomical distribution, half were limited exclusively to the lower limbs. These data suggest that at least half of the patients with unknown HIV status were likely to be actually HIV infected. A Kenyan study has shown that, currently, endemic and HIV-related exhibit similar clinical presentation and natural history [25]. In an Ugandan study of 197 adults with HIV and KS, on the contrary, 55 percent were women; they were less likely to have lower limb lesions and more likely to have facial lesions than men. The authors suggested that gender affects the pathophysiology of KS [26].

## 5. Conclusion

The present study shows a relatively high discordance rate between the clinical and the biopsy-proven diagnosis of KS, indicating that “violaceous” skin lesions in an HIV endemic population warrant a biopsy. The relatively high histological discordance between the pre- and post-immunohistochemical diagnosis of KS warrants HHV-8 immunophenotyping to ascertain the diagnosis.

## Figures and Tables

**Table 1:** Age distribution by gender, HIV serostatus, and Kaposi's sarcoma.

	Male	Female	*t*	*P*
HIV seropositive	41.6 ± 12.6 [41.0]*	40.5 ± 10.7 [38.0]	0.46	0.15
HIV status unknown	41.7 ± 18.0 [43.0]	41.1 ± 18.8 [39.0]	0.21	0.84
Biopsy-proven KS	41.1 ± 14.1 [40.0]	42.2 ± 15.7 [39.5]	0.54	0.59

* Values are mean ± SD [median].

**Table 2:** Distribution by anatomical sites of Kaposi's sarcoma: HIV-positive *versus* unknown serostatus.

Site	Males HIV positive *n* (%)	Females HIV positive *n* (%)	Total HIV positive *n* (%)	Males HIV status unknown *n* (%)	Females HIV status unknown *n* (%)	Total HIV status unknown *n* (%)
Head	7 (8.8)*	4 (7.0)*	11 (8.0)^†^	9 (23.7)*	9 (24.3)*	18 (24.0)^†^
Trunk	5 (6.3)	1 (1.8)	6 (4.4)	3 (7.9)		3 (4.0)
Upper limbs	4 (5.0)	1 (1.8)	5 (3.6)	3 (7.9)	3 (8.1)	6 (8.0)
Lower limbs	34 (42.5)	23 (40.3)	57 (41.6)	17 (44.7)	18 (48.6)	35 (46.7)
Perineal	1 (1.3)	4 (7.0)	5 (3.6)	1 (2.6)	2 (5.4)	3 (4.0)
Disseminated	29 (36.2)	24 (42.1)	53 (38.7)^†^	5 (13.2)	5 (13.5)	10 (13.3)^†^
Unknown	4 (4.8)**	20 (26.0)**	24 (14.9)**	14 (36.8)**	11 (22.9)**	25 (25.0)**

Total	84	77	161	52	48	100

*% of the total number of known anatomical sites; ** % of the total number of cases; ^†^ statistically significant 95% confidence intervals (CIs).

**Table 3:** Distribution by stage of Kaposi's sarcoma: HIV-positive *versus* unknown serostatus.

Stage	Males HIV positive *n* (%)	Females HIV positive *n* (%)	Total HIV positive *n* (%)	Males HIV status unknown *n* (%)	Females HIV status unknown *n* (%)	Total HIV status unknown *n* (%)
Early patch	45 (53.6)	35 (45.5)	80 (49.7)	21 (40.3)	15 (31.3)	36 (36.0)
Plaque	9 (10.7)	17 (22.1)	26 (6.1)	7 (13.5)	7 (14.5)	14 (14.0)
Nodular	30 (35.7)	25 (32.4)	55 (34.2)	24 (46.2)	26 (54.2)	50 (50.0)

Total	84	77	161	52	48	100

**Table 4:** Biopsy-proven non-Kaposi's sarcoma skin lesions and HIV status.

Pathology	HIV seropositive *n* (%)	HIV serostatus unknown *n* (%)	Total *n* (%)
Pyogenic granuloma	24 (27.6)	39 (27.5)	63 (27.5)
Seborrheic keratosis	29 (33.3)	22 (15.5)	51 (22.3)
Haemangioma	4 (4.6)	33 (23.2)	37 (16.2)
Fibrous histiocytoma	4 (4.6)	21 (14.8)	25 (10.9)
Drug reaction vasculitis	5 (5.7)	1 (0.7)	6 (2.6)
Melanocytic melanoma	3 (3.4)	2 (1.4)	5 (2.2)
Varia	18 (20.8)	24 (16.9)	42 (18.3)

Total	87	142	229

**Table 5:** Histological diagnostic discordance before immunohistochemistry.

Pathology	Overdiagnosis of KS *n* (%)	Underdiagnosis of KS *n* (%)	Total discordance rate *n* (%)
Seborrheic keratosis	8 (29.6)	7 (38.9)	15 (33.3)
Fibrous histiocytoma	9 (33.3)	3 (16.7)	12 (26.7)
Pyogenic granuloma	5 (18.5)	5 (27.8)	10 (22.2)
Drug reaction vasculitis	3 (11.1)	3 (16.7)	6 (13.3)
Haemangioma	2 (7.4)		2 (4.4)

Total	27	18	41/490 (9.2)

**Table 6:** Differential diagnosis of Kaposi's sarcoma.

Patch stage		Plaque stage	Nodular stage	Aggressive late stage
Early macular	Late macular			

Pyogenic granuloma	Well-differentiated angiosarcoma	Angioendotheliomatosis	Pyogenic granuloma	Desmoplastic malignant melanoma
		Acroangiodermatitis	Bacillary angiomatosis	Fibrosarcoma

Atrophic histiocytoma	Progressive lymphangioma	Benign-disseminated angioproliferation	Spindle cell hemangioma	Leiomyosarcoma
			Kaposi-form hemangioendothelioma	Monophasic synoviosarcoma

Benign lymphangioendothelioma	Microvenular hemangioma	Dermatomyofibroma	Cutaneous leiomyosarcoma	
	Granuloma annulare	Targetoid hemosideric angioma	Cutaneous angiosarcoma	
			Hypertrophic scar	
			Dermatofibroma/sarcoma protuberans	
			Neurofibroma	
			Spindle cell melanoma	
			Fibrous histiocytoma	
